# Developing theoretically underpinned primary care resources for patients with asthma: an exemplar from the IMP^2^ART trial

**DOI:** 10.1017/S1463423624000197

**Published:** 2024-09-20

**Authors:** Atena Barat, Kalina Czyzykowska, Kirstie McClatchey, Tracy Jackson, Liz Steed, Jessica Sheringham, Viv Marsh, Elisabeth Ehrlich, Noelle Morgan, Vicky Hammersley, Steve Holmes, Brigitte Delaney, Stephanie J.C. Taylor, Hilary Pinnock, for the IMPART Programme Group

**Affiliations:** 1 Wolfson Institute of Population Health, Barts and the London School of Medicine and Dentistry, Queen Mary University of London, London, UK; 2 Asthma UK Centre for Applied Research, Usher Institute, The University of Edinburgh, Edinburgh, UK; 3 Department of Applied Health Research, University College London, London, UK; 4 The Park Medical Practice, Shepton Mallet, UK; 5 Severn School of Primary Care, Health Education England (South West), Bristol, UK; 6 School of Health and Related Research, University of Sheffield, Sheffield, UK

**Keywords:** asthma, asthma education, patient education, patient resources

## Abstract

**Aim:**

This article reports on the development of patient resources for the IMPlementing IMProved Asthma self-management as RouTine (IMP^2^ART) programme that aimed to encourage patients to attend asthma reviews (invitation letters), encourage patients to enquire about asthma action plans (posters), and equip patients with the knowledge to manage their asthma (information website).

**Background:**

To improve supported asthma self-management in UK primary care, the IMP^2^ART programme developed a whole-systems approach (patient resources, professional education, and organisational strategies).

**Methods:**

Linked to behaviour change theory, we developed a range of patient resources for primary care general practices (an information website, invitation letters to invite patients for asthma reviews, and posters to encourage asthma action plan ownership). We elicited qualitative feedback on the resources from people living with asthma in the UK (*n* = 17). In addition, we conducted an online survey with volunteers in the UK-wide REgister for Asthma researCH (REACH) database to identify where they source asthma information, whether their information needs are met, and what information would be useful (*n* = 95).

**Findings:**

Following feedback gathered from the interviews and the online survey, we refined our patient resources for the IMP^2^ART programme. Refinements included highlighting the seriousness of asthma, enhancing trustworthiness, and including social support resources. We also made necessary colour and formatting changes to the resources. In addition, the patient resources were updated following the COVID-19 pandemic. The multi-stage development process enabled us to refine and optimise the patient resources. The IMP^2^ART strategy is now being tested in a UK-wide cluster RCT (ref: ISRCTN15448074).

## Introduction

There are approximately 5.4 million people living with asthma across the United Kingdom (UK) (Asthma UK, 2020) resulting in around 6.3 million primary care consultations and 100 000 hospital admissions per year (Mukherjee *et al.*, 2016). A recent meta-review found that supported asthma self-management can reduce hospitalisations, accident and emergency attendances, and unscheduled care consultations (Pinnock *et al.*, 2017). Further, supported self-management can improve markers of asthma control and quality of life across a range of demographic, cultural, and healthcare settings (Pinnock *et al.*, 2017). Core components of supported asthma self-management include a personalised asthma action plan, regular clinical review, and appropriate patient education (Hodkinson *et al.*, 2020).

Despite strong evidence of its benefits (Pinnock *et al.*, 2017), and recommendations in national and international asthma guidelines (BTS/SIGN, 2019; Global Initiative for Asthma, 2022), supported asthma self-management is poorly implemented. For example, only half of respondents (52%) to a survey conducted by Asthma UK owned an asthma action plan (Asthma UK, 2020), whilst in our review of clinical records only 6% of people with asthma had a record of being provided with an action plan (Newby *et al.*, 2017). Further, according to the annual Asthma UK survey (Asthma UK, 2020), almost 30% of respondents had not attended an annual asthma review in which basic asthma care can be provided and supported self-management discussed.

To promote asthma self-management and support action plan implementation, understanding the needs of people with asthma is pivotal. A recent systematic review exploring barriers and facilitators of effective asthma self-management identified that patients expressed a need for information, especially about asthma control, triggers, and medication (Miles *et al.*, 2017). These findings were relatively universal including among those with low health literacy and those from ethnic minorities, who actively sought information often from lay sources (Miles *et al.*, 2017). Similarly, a survey from Australia on information needs of those with asthma showed that a third of respondents had outstanding information needs (Kong *et al.*, 2013) on similar topics.

In response to the poor implementation of supported asthma self-management in UK primary care, the IMPlementing IMProved Asthma self-management as RouTine (IMP^2^ART) programme was developed (McClatchey *et al.*, 2023). IMP^2^ART aims to develop and evaluate a whole-systems implementation strategy comprising of resources for patients (e.g. information for patients, invitation letters showing the importance of asthma reviews, promotional materials that highlight the importance of asthma action plans), professional training, and organisational resources. This paper reports on the design and development of theoretically informed patient resources that can be used in primary care general practices to help provide information, encourage attendance at annual reviews, and encourage action plan ownership for those living with asthma.

## Methods

**Figure 1.**
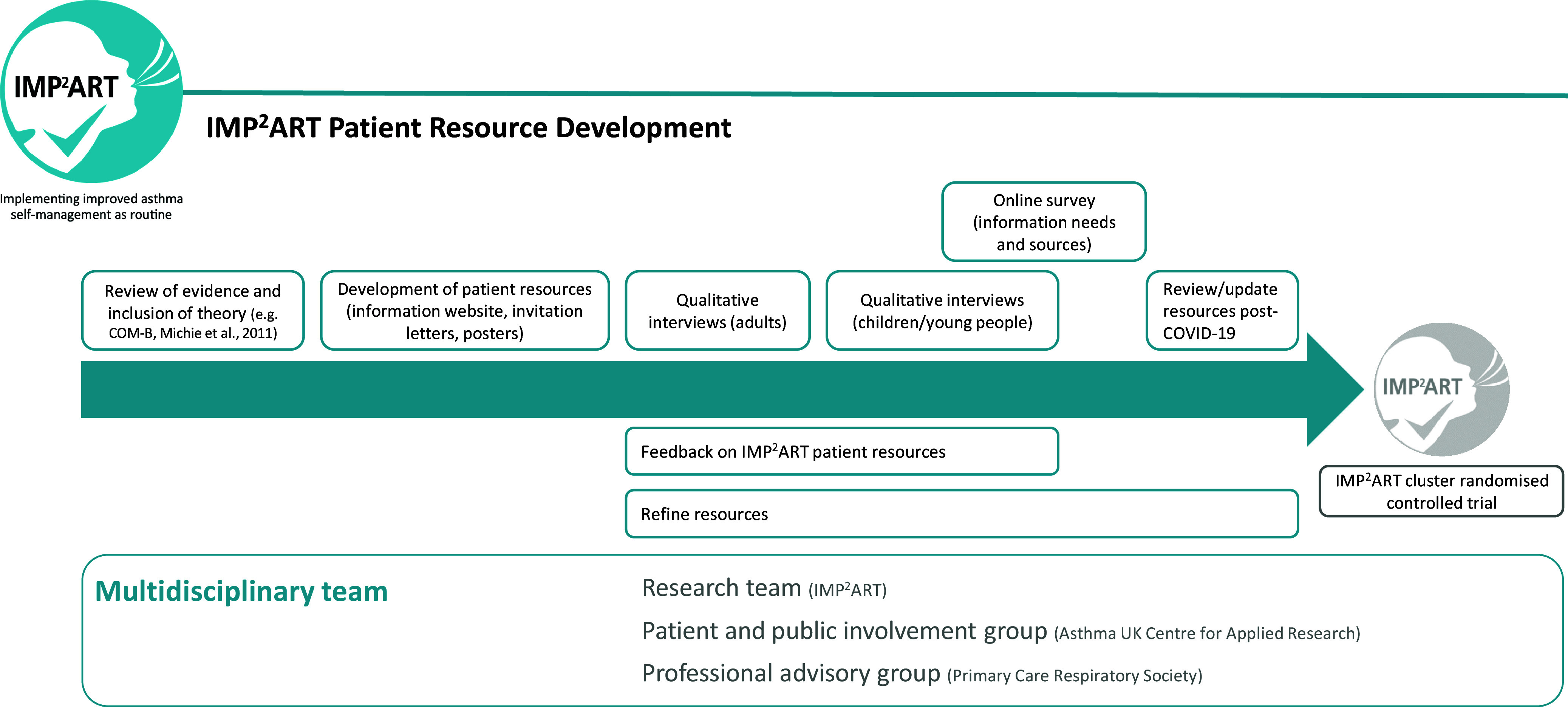
Development phases of the IMP^2^ART patient resources.

The IMP^2^ART patient resources were developed between late-2018 and late-2020 (see Figure 1) in line with O’Cathain *et al.*’s (2019) guidance on how to develop complex interventions to improve health and healthcare. The IMP^2^ART team developed resources with our Asthma UK Centre for Applied Research (AUKCAR) Patient and Public Involvement (PPI) group (‘PPI colleagues’), and our Professional Advisory Group (‘Professional colleagues’), which consisted of general practitioners (GPs) and nurses from the Primary Care Respiratory Society (PCRS). Table 1 maps the development of the patient resources to the guidance.

**Table 1.** Framework of actions for intervention (patient resources) development (O’Cathain *et al.*, 2019)

Action	Application to the development of patient resources within the IMP^2^ART programme
Plan the development process	Supported self-management for asthma, which includes patient education, regular review, and personalised asthma action plan provision, has been recommended by guidelines for 30 years (British Thoracic Society *et al.*, 1990). Evidence from 27 systematic reviews (270 RCTs), concluded that supported self-management reduces hospitalisations, accident and emergency attendances, unscheduled consultations, and improves markers of asthma control and quality-of-life (Pinnock *et al.*, 2017). However, action plan provision is poorly implemented (only 52% of those surveyed) (Asthma UK, 2020), and in the National Review of Asthma Deaths, 77% of those who died did not have an asthma action plan (Royal College of Physicians, 2014). Whilst patient education, professional training, and organisational support are all essential to support self-management, they are rarely effective in isolation (Taylor *et al.*, 2014). Therefore, within the IMP^2^ART programme, we aimed to develop patient resources within a whole-systems implementation strategy (https://www.ed.ac.uk/usher/imp2art).
Involve stakeholders, including those who will deliver, use and benefit from the intervention	We enlisted the expertise of the AUKCAR Patient and Public Involvement group (‘PPI colleagues’). This included those with lived experience supported by researchers with expertise in facilitating PPI groups. We formed a Professional Advisory Group (PAG) of GPs and nurses from the Primary Care Respiratory Society (PCRS). Both PPI and PAG meetings took place at regular intervals over the course of the development.
Bring together a team and establish decision-making processes	The IMP^2^ART team consists of primary care academics, general practitioners, health psychologists, nurses, PPI and PAG colleagues. We discussed widely at weekly team meetings with all views welcomed. Senior members of the IMP^2^ART team took final decisions on patient resource content to meet majority preferences, ensure adherence to guideline recommendations, and promote supported asthma self-management implementation.
Review published research evidence	Patient resources were to be aligned with BTS/SIGN (2019), NICE (2021), and GINA (2022) global guidelines on the management of asthma. All the guidelines recommend supported self-management and agree that this should include advice on adherence to maintenance treatment, recognising deterioration, action to be taken, and routine reviews. Where there were discrepancies (such as GINA’s increased emphasis on first-line use of combined ‘Maintenance and Reliever Therapy’ which is not recommended by UK guidelines), on the advice of the PAG, we followed UK guidelines. General practice staff recognise that patients want information about managing their asthma, but consider that a limited range of resources made it difficult to find action plans and information tailored to individual patients. They also note that encouraging patients to attend for routine asthma reviews is challenging (Morrow *et al.*, 2017). For patients, self-management education (with an action plan) has particular value early after diagnosis when coping strategies are beginning to form, and after an attack when existing management has clearly failed (Daines *et al.*, 2020).
Draw on existing theories	The COM-B (capability, opportunity, motivation, behaviour) framework for understanding behaviour (Michie *et al.*, 2011) and the Behavior Change Technique Taxonomy (Michie *et al.*, 2013) were used in the development of all IMP^2^ART resources, and all resources were designed to incorporate relevant behaviour change techniques.
Articulate programme theory	A full programme theory including a logic model for the IMP^2^ART programme has been developed (Steed *et al.*, 2023). Evaluation of the programme theory for the patient resources will be explored in a process evaluation of the IMP^2^ART cluster-RCT.
Undertake primary data collection	Following the design of the patient resources (detailed in the ‘design and refine the intervention section’), we tested feasibility of the resources and sought users’ perceptions. 17 interviews were conducted (with adults and children/young people) following the initial design of the IMP^2^ART developed patient resources. An online survey with 95 individuals was conducted to ensure we captured well-used information sources for the IMP^2^ART patient information website and included information where gaps where identified.
Understand context	Primary care is made up of varying demographics (e.g. ages, ethnic groups, socioeconomic groups) and varying geography (e.g. rural, urban). For general practices in England, the Quality and Outcomes Framework determines performance-related payments (NHS Confederation, 2003). General practices use different systems, therefore we designed aspects of the IMP^2^ART patient resources (e.g. invitation letters) so they could be adapted by the practice.
Pay attention to future implementation of the intervention in the real world	IMP^2^ART is a programme of implementation research. The resources were developed explicitly for implementation in routine clinical practice within the context of a UK-wide cluster randomised implementation trial (https://www.isrctn.com/ISRCTN15448074). The patient resources were designed to be simple and accessible for ease of use in primary care. The process evaluation will consider future use of the intervention, ‘scale up’ and sustainability in real-world contexts.
Design and refine the intervention	Linked to behaviour change techniques (Michie *et al.*, 2013), and considering health literacy, the IMP^2^ART team with PPI and professional colleagues designed the following patient-facing resources: a website containing information about asthma and a variety of asthma action plans. templates for letters that general practices can use to invite patients for their annual review, remind patients about missed appointments, and invite patients to a review following unscheduled care (all highlighting the importance of asthma action plans). Posters that general practices can use to encourage patients to ask about an action plan if they do not have one. Following the primary data collection (interviews with adults and children/young people, and the online survey) we refined the patient resources. The resources were also reviewed and updated following the outbreak of the COVID-19 pandemic to meet additional patient information needs (McClatchey *et al.*, 2021).
End the development phase	We finalised resources for use in the IMP^2^ART cluster-RCT.

### Design of the IMP^2^ART patient resources

Prior research (Miles *et al.*, 2017; Morrow *et al.*, 2017; Daines *et al.*, 2020) has highlighted that patients want more information about managing their asthma, and that encouraging patients to attend for routine asthma reviews can be challenging. Considering this with the low uptake of asthma action plans (Asthma UK, 2020), and with the advice of the patient and professional colleagues, three resources were conceived: a patient information website, invitation letters for general practices to invite patients for asthma reviews, and posters to encourage asthma action plan ownership. To develop the resources, we utilised behaviour change techniques (the active component of an intervention designed to change behaviour) from the Behavior Change Technique Taxonomy (Michie *et al.*, 2013). The three resources are outlined below.

#### Patient information website

For the patient information website, we aimed to find existing relevant online asthma information from trusted asthma sources (e.g. the charities Asthma UK and the British Lung Foundation (now merged as Asthma and Lung UK), the European Lung Foundation, My Lungs my Life, and the NHS) and house all the information topics on one site with links to the original sources. The information included a variety of topics that covered, for example: general asthma information (e.g. what is asthma); annual asthma reviews; triggers; living with asthma (e.g. exercise, sleep, travel etc.); asthma attacks; coping with asthma and emotions; children and young people; asthma management (e.g. asthma action plans – where we considered various languages, literacy, and age groups); and inhaler technique videos.

#### Invitation letters

The IMP^2^ART team designed three invitation letter templates that general practices could tailor and send to patients to: 1) invite patients for their annual review, 2) remind patients about missed review appointments, 3) invite patients to a review following unscheduled care. Invitation letters were designed to include behaviour change techniques identified from the Behavior Change Technique Taxonomy (Michie *et al.*, 2013) including *‘information about health consequences’,* for example, that asthma is a variable condition and self-management can reduce symptoms and the risk of an asthma attack, and a *‘social comparison’*, for example, that one in three people in the UK have an asthma action plan.

#### Posters for use in primary care practices

We designed two posters for use in general practices to encourage and prompt conversations around asthma action plan ownership, one for noticeboards and a digital version for display screens. Both poster types contained the same text content. Posters were also designed in line with behaviour change techniques (Michie *et al.*, 2013), and included *‘information about health consequences’*, for example, that asthma causes 6.3 million GP consultations per year, and a *‘social comparison’* for example that one in three people in the UK have an asthma action plan.

### Two rounds of feedback and refining the patient resources

We conducted semi-structured qualitative interviews with adults in the UK living with asthma (in February–March 2019) to obtain initial feedback on the prototype IMP^2^ART resources. Participants were purposively recruited from six demographically diverse general practices (e.g. patient list size, socioeconomic status). Eligible patients included those over 18 years old with ‘active asthma’ (defined as having a coded diagnosis and received pharmacological treatment within the previous year (NHS Confederation, 2003)), with the capacity to provide informed consent and take part in the interview. GPs could exclude participants in the event of substantial comorbidity (e.g. dementia), illness, or residential care.

Prototype versions of the resources were sent to participants in a pdf format. The website pdf listed the source of the online information (e.g. Asthma UK) and was broken down into topic areas (e.g. triggers) with subheadings (e.g. pollen). The topic guide asked participants what thoughts they had on the resources, and what feedback they had (if any). Following giving participants information about the study and obtaining informed consent, interviews were conducted via telephone and were carried out by KM (a health psychologist) and KS (a medical student) and lasted 20 to 40 min. Participants were unknown to the researchers.

Following refinements of the resources based on recommendations from round one interviews, we realised that many of the resources were directed at children and therefore conducted a second round of semi-structured qualitative interviews and focus groups with children and young people. They were purposively recruited via a general practice and the SPEAK Asthma network (a group enabling children and young people living with asthma to get involved in research at the Asthma UK Centre for Applied Research). Eligible patients included those 6–16 years old with ‘active asthma’ and having the capacity to provide informed consent or take part in the interview. Interviews and focus groups were conducted face-to-face by KM between July and September 2019 after informed consent was obtained, and lasted between 15 and 35 min in duration. Interviews from both phases were audio-recorded, transcribed verbatim, and analysed using Framework Analysis (Ritchie & Spencer, 1994) supported by NVivo 11.

### Sense-checking resources for the patient website (quantitative survey)

We conducted an online survey, to ensure we captured well-used information sources for the website and included information where gaps where identified. The survey included both open and closed questions that were designed in line with the Practical Reviews in Self-Management Support (PRISMS) taxonomy (Pearce *et al.*, 2016). The survey questions included demographic information; perception of asthma symptoms; where information about asthma is sourced (e.g. from healthcare professionals, websites); whether information needs are met; and what information would be useful for patients. Survey participants were recruited using the REgister for Asthma researCH (REACH) recruitment database. REACH was a secure database that people living with asthma (aged 16 years and over) in the UK were able to register their information, in order to be informed about research studies. Participants signed up to the database were emailed information about the survey and instructions on how to participate (if they were over 16 years old with ‘active asthma’). Following obtaining informed consent, data were collected between August and September 2019. Survey data were anonymised and analysed using descriptive statistics in SPSS version 24.

## Results

### Feedback on the patient resources (qualitative interviews with adults)

A total of ten participants completed interviews (seven female; age range 38–75 years, all located in Scotland), one of whom also provided feedback on the resources via email, and a further two individuals provided feedback on the resources via email alone. Three themes were identified and are described below.

#### Patient information website

Almost all participants had positive perceptions of the prototype website, finding the content clear, concise, and educational. Participants generally agreed that the website was comprehensive, as there was useful information across a wide range of topics which covered various demographics (e.g. children and adults).

*‘The “for kids” one I thought was extremely good, I really liked that… It’s making it child-friendly, which I thought was a really nice thing’. (Participant 5, female)*

Some participants viewed the website as a good source to learn more about their condition and even gained new knowledge.

*“I didn’t realise that – I don’t drink a lot – but I didn’t realise until I read this that alcohol can affect your asthma…” (Participant 8, male)*

Trustworthiness was discussed as key to patient information. The NHS website was regarded as an especially reliable source, being the primary online resource for some participants. Participants voiced that they trust sources recommended by their healthcare professional (e.g. GP, pharmacist).

*“The NHS website I think is very good to check up on ailments and things, and the information they give, I trust that quite a lot. I think there are some websites and things that are a bit… not trustworthy.” (Participant 3, female)*

In terms of recommendations for the website, a participant suggested to include an opening description for the website, and one participant suggested the inclusion of social support networks for people with asthma, as it was something she found valuable when managing her condition.

#### Invitation letters

Information included in the letters was viewed positively by participants in terms of simplicity, clarity, and the amount of detail covering everything patients would need to know before attending a review appointment.

*“It’s not long-winded, it’s reasonably short, to-the-point, and it’s clear and easy to understand.” (Participant 6, female)*

The significance of letters in asthma care was recognised, as participants appreciated receiving such letters particularly after experiencing an asthma attack.

*“I would be quite pleased that somebody was following up on the attack and making sure that I was having the right treatment and doing the proper things to help prevent having another one.” (Participant 8, male)*

Most participants found the IMP^2^ART invitation letters similar to the letters they already receive from their practices, although more detailed and with the explicit mention of action plans. In contrast, a few interviewees mentioned the level of detail in the letters was ‘too wordy’ and ‘dense’. Furthermore, all participants agreed that both annual review and post-asthma attack letters would have a positive effect on attendance. Some interviewees suggested that the letters should emphasise the importance of reviews by highlighting (in a sensitive manner) the negative health consequences of not attending reviews.

*“…say that this review is to help you get over your asthma attacks, to miss out on this review may involve you suffering more. Without being bullyish.” (Participant 8, male)*

Participants offered a variety of suggestions for emphasising the necessity of having a post-attack review, such as adding colour, boldness, and making the important information larger and more strongly worded.

#### Posters for use in primary care practices

Participants indicated that the information on both the noticeboard and digital poster was clear and easily comprehensible, some attributing this to the simple language.

*“It’s in very straightforward English, it’s not oodles and oodles of information, and it’s nice short concise sentences.” (Participant 1, female)*

Views varied on visual aspects of posters such as layout, graphics, colour, text size, and blank space. Some of the participants thought that the noticeboard poster was eye-catching due to its colour and brightness while others thought that it was cramped and too busy. Some interviewees, however, described the digital posters which had less imagery as ‘*dull’*, and not as eye-catching as the noticeboard posters but conversely thought they were more effective in presenting the information as they had more free space around the text. They believed that digital posters would be noticed more because people nowadays pay more attention to electronic screens. Participants agreed that the posters would have some degree of success in encouraging patients to enquire about or use action plans. They assumed that the information on the benefits of action plans (e.g. reducing symptoms, improving quality of life) would spark interest in asthma patients.

*“…it’s suggesting it [an action plan] can reduce the symptoms and increase your quality of life then obviously you would be interested, wouldn’t you?” (Participant 10, male)*

Other participants, through recalling their own experiences of sitting in waiting rooms, stated that unwell patients may be unlikely to pay attention to any posters.

*“I think people in that situation… I know from when I was at [a hospital for treatment], that you’re so focused on your own thing, it’s not the point that you’re maybe ready to absorb information like that, the visual cues.” (Participant 9, female)*

The majority of suggestions about the poster centred around its design, such as removing images to make the noticeboard poster less busy and replacing them with asthma-related information (e.g. a helpline number). Further, participants suggested stronger wording to emphasise the importance of asthma action plans.

*“It says “asthma causes 6.3 million GP consultations a year” but it’s not saying that, you know, asthma is dangerous…what it’s not saying here is that actually, you know, an action plan could actually save your life.” (Participant 5, female)*

### Refinements to the patient resources following the qualitative interviews with adults

Following the qualitative exploration, we designed the next version of the patient information website with University of Edinburgh website developers. The IMP^2^ART team and PPI colleagues agreed on visual options for the website (including both desktop and mobile versions). We included a clear descriptor on the homepage detailing the purpose of the website and that the site had been designed by a team of researchers and patients at the Asthma UK Centre for Applied Research. This descriptor was designed to enhance trustworthiness (which was highlighted by participants in the interviews as being an important factor), and we also added the university logo to the website footer. Through feedback from the PPI group, accessibility was considered, for example, images descriptions were added where necessary and embedded videos included subtitles.

For the invitation letters, we made minor formatting changes, including making some content bold to highlight the importance of the review, and added a sentence that asked patients to bring their asthma action plan to their review. We did not respond to some suggestions, for example, to add colour, as we learnt from qualitative data collection that general practices rarely use colour printing (Morrow *et al.*, 2017). We refined the posters to ensure that the images were clear. We also changed the opening statement from ‘Asthma causes 6.3 million GP consultations a year’ to ‘Asthma causes 121 000 A&E visits each year’. This was based on feedback to highlight the seriousness of asthma.

### Feedback on the refined patient resources (qualitative interviews with children and young people)

A total of seven children and young people (five female; age range 11–15 years, located in both England and Scotland) participated in interviews or focus groups, and we found that participants were positive about the patient information website (although they rarely utilised the internet for information about asthma). One focus group participant mentioned that the colours and icons of the website looked *“very NHS-ey”* which another participant agreed and said it *“associates it with…the healthcare system… it associates you into thinking it’s a good reliable website”.* The participants did not have any feedback about the invitation letters, and although most were positive about the posters it was recommended to make the colours stand out more.

### Refinements to the patient resources following the qualitative interviews with children and young people

**Figure 2.**
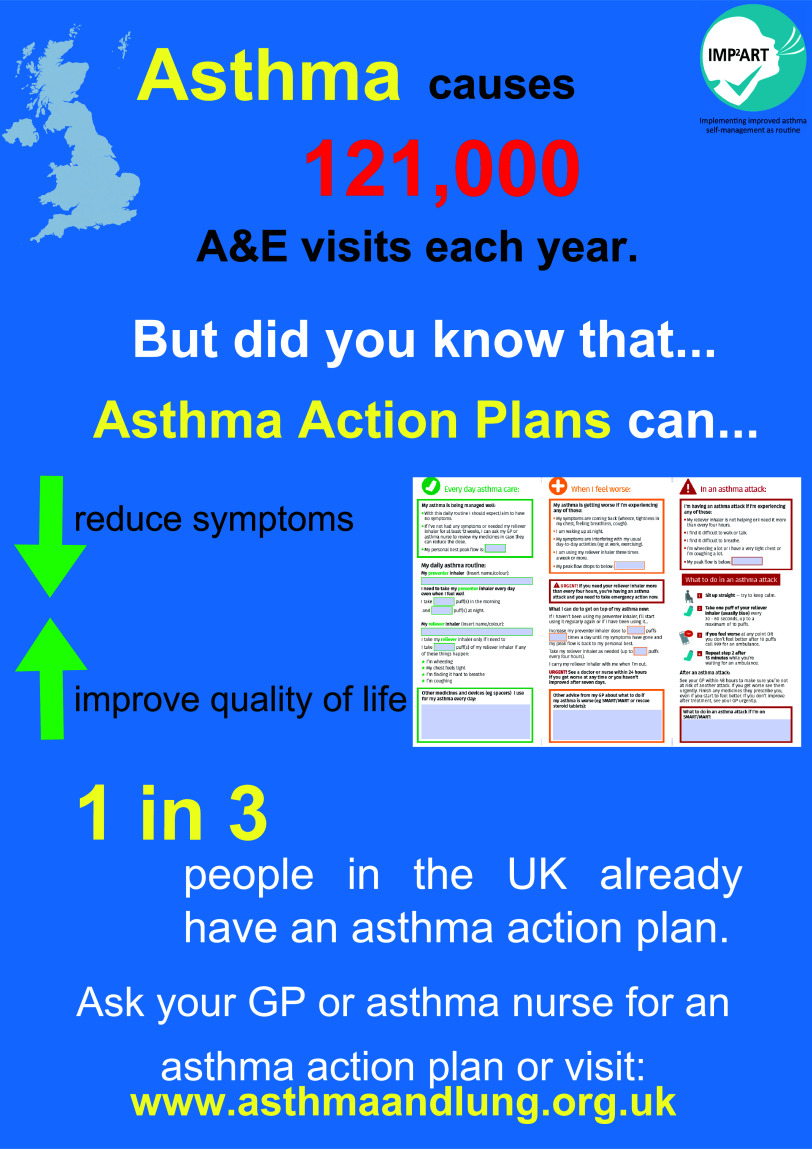
Finalised version of the noticeboard poster for use in general practices to encourage asthma action plan ownership.

We made minor changes to some of the colours on the poster to stand out. Figure 2 displays the finalised version of the noticeboard poster.

### Sense-checking resources for the patient website (online survey)

Of the total 151 eligible participants invited to participate, 95 completed the online survey (62.9% response rate). Participant characteristics can be found in Table 2.

**Table 2.** Online survey participant characteristics

Participant characteristics		*n*	*%*
Age group (years)	16–25	20	21.1
	26–35	15	15.8
	36–45	21	22.1
	46–55	13	13.7
	56–65	18	18.9
	65 and over	8	8.4
Gender	Male	29	30.5
	Female	65	68.4
	Non-binary	1	1.1
Duration of asthma diagnosis	Less than a year	2	2.1
	1–10 years	15	15.8
	More than 10 years	78	82.1
Perceived asthma severity	Very mild	6	6.3
	Mild	24	25.3
	Moderate	40	42.1
	Severe	19	20
	Very severe	6	6.3

Fewer than half of the respondents had an asthma action plan (*n* = 45; 47.4%), and of those, 82% received the action plan from a healthcare professional. Most respondents had a regular asthma review at their general practice (*n* = 75; 78.9%), mostly with a nurse (*n* = 68; 90.7%), though GPs (*n* = 6; 8.0%) and pharmacists (*n* = 1; 1.3%) also conducted reviews.

#### Information provision

Fewer than half of the respondents felt that all or a lot of their asthma information needs were met (*n* = 44; 46.3%), and only half (*n* = 51; 53.7%) felt there was adequate information available from their general practice explaining asthma and how to manage it. Over half of the sample (*n* = 53; 55.8%) had not received any resources to help with asthma management skills (e.g. information about inhaler technique). Further, social support was recommended by a healthcare professional to only 2.1% of participants (*n* = 2).

#### Information sources and usage

Most participants (*n* = 72; 75.8%) would contact their general practice if they had any questions about their asthma. Other information sources included websites (*n* = 58; 61.1%) (mostly Asthma UK (*n* = 31), and the NHS (*n* = 22)); the Asthma UK helpline (*n* = 25; 26.3%); pharmacists (*n* = 24; 25.3%); the emergency department (*n* = 10; 10.5%); and the NHS 24 helpline (*n* = 8; 8.4%). Most respondents had not used resources to encourage them to take medication for their asthma (*n* = 78; 82.1%). Less than a third of respondents used resources to monitor asthma symptoms (*n* = 29; 30.5%), and of those, 62.1% used mobile applications, and 34.5% used a diary to monitor symptoms. Most of the sample did not use any resources to help them to prepare for consultations with their healthcare provider about their asthma (*n* = 79; 83.2%). Further, most of the sample did not use any information to support the day-to-day management of their lifestyle (*n* = 68; 71.6%).

#### Information recommendations

Those who felt that there was not adequate information available to them from their general practice were asked what information would be helpful. The most requested information included asthma leaflets (*n* = 6), advice on asthma treatment (e.g. inhalers, how to use them, and side effects) (*n* = 6), provision of an action plan (*n* = 6), information on how asthma happens and diagnoses information (*n* = 3), information about severe asthma (*n* = 4), and support groups available (*n* = 2). Respondents also suggested there should be posters about asthma in general practices (*n* = 3), and further information available online (*n* = 2) including *‘a repository of information available online to patients’*.

In an optional open response question, respondents were able to describe the information about asthma that they most needed. Treatment and medication information (including how to use inhalers) (*n* = 28) and information about asthma management including how to manage symptoms and when to call for help (*n* = 29) were the most stated needs. Additionally, a number of respondents wanted further information on asthma triggers (*n* = 4) information about asthma generally, for example, why it happens, how it affects the body, and the long-term prognosis (*n* = 3) and day-to-day living with asthma, for example, employment, sleep, and travel (*n* = 5).

### Refinements to the patient resources following the online survey

The survey supported our rationale to provide various resources for patients, for example, an information website and posters about asthma. In terms of website content, the survey ensured that we included suggested topics and relevant sources of information for those living with asthma in the UK. For example, we added a dedicated social support section, which is linked to various trusted asthma relevant social support forums (e.g. Asthma UK and British Lung Foundation).

### Refinements following the COVID-19 pandemic

During the COVID pandemic, we reviewed our IMP^2^ART patient resources. We conducted a rapid review of COVID-19 information for those living with asthma (McClatchey *et al.*, 2021) and added a section on the patient information website for COVID-19 and asthma. We also collaborated with PPI and professional colleagues to create additional videos for the website on topics including: ‘Patient’s tips for managing your asthma’; ‘Get the best from your asthma review’; and ‘Working together with patients’.

Additionally, we changed the wording of the invitation letters to reflect that appointments may be conducted remotely, and we added an image on the letter to reflect a remote appointment (a smartphone and computer screen).

### A dynamic and iterative process

The process of developing the finalised IMP^2^ART patient resources followed key principles of intervention development, that it is dynamic, iterative, creative, and open to change (O’Cathain *et al.*, 2019). We have continued to identify new content for the information website as it became available throughout the development process – for example, reflecting the topic of climate change and metered dose inhalers.

## Discussion and conclusion

We developed a suite of resources for patients (information website, invitation letters, and posters) that general practices in the implementation arm of the IMP^2^ART cluster-RCT could use to support asthma self-management. We followed a systematic approach to intervention development (O’Cathain *et al.*, 2019), and the resources were initially developed in line with behaviour change theory and adapted according to feedback from those currently living with asthma. Further, this paper adds to the available knowledge on the self-management educational needs of asthma patents in the UK.

Prior research and the current online survey exploration shows that asthma information needs are often not met (Kong *et al.*, 2013; Miles *et al.*, 2017), and the IMP^2^ART patient website aims to address this gap. The IMP^2^ART patient website was adapted during its development to ensure trustworthiness following the qualitative feedback we received. This may have a positive effect on trust and credibility, as a review exploring web-based health information found that the authority of the owner can positively impact trust and credibility (Sbaffi & Rowley, 2017). By ensuring that the information aligned with UK guidelines (BTS/SIGN, 2019; NICE, 2021), we reduced the risk of patients receiving conflicting advice.

Both the patient website and the invitation letters highlight the importance of asthma reviews, which may improve on the current figures of non-attendance at asthma reviews (approximately 30% (Asthma UK, 2020)). Further, there are suboptimal levels of asthma action plan ownership in the UK (Newby *et al.*, 2017; Asthma UK, 2020), and the patient information website, the invitation letters, and the posters for use in primary care practices may encourage asthma action plan ownership. With regard to the posters, following the qualitative interviews, we made refinements to the posters to highlight the seriousness of asthma. Recent qualitative work with those living with asthma in the UK has found that perceptions about how serious asthma can be are variable, and that seriousness may not be apparent until an admission to hospital or the experience of a severe attack (Apps *et al.*, 2019). By highlighting the seriousness of asthma, as suggested by our participants currently living with asthma, this may encourage asthma self-management.

### Strengths and limitations

A major strength of the work was following guidance on how to develop complex interventions to improve health and healthcare (O’Cathain *et al.*, 2019) and the use of a mixed-method design to elicit feedback from participants (e.g. interviews with both adults and children/young people, and a survey). An additional strength was directly involving those currently living with asthma in the UK, who will be receiving support from their primary care provider. Although we only interviewed ten adults and seven children, we also had input from 95 respondents to the survey and interpretation and advice was provided by the PPI colleagues (*n* = 4). Further, healthcare professionals were not interviewed or surveyed as part of patient resource development, although the IMP^2^ART team includes healthcare professionals, and the Professional Advisory Group who contributed to the design and development of the resources is made up of GPs, nurses and pharmacists currently working in primary care.

A further strength is that the iterative methodology allowed for adaptation, for example, adding information to the website about the pandemic and updating all the resources to consider remote consulting. This adaptability has continued as we have added new resources to reflect topical issues such as the switch to dry powder devices in response to zero carbon initiatives. In the future, a change in resources may be needed if UK guidelines change to recommend ‘as required’ combination inhalers.

### Practice implications

The IMP^2^ART strategy (incorporating the patient resources, as well as professional training and organisational resources) is now being tested in a UK-wide cluster RCT (ref: ISRCTN15448074), evaluating implementation (action plan ownership) and health outcomes (unscheduled care). We will track patient information website usage during the trial using Google Analytics, and the process evaluation (Sheringham *et al.*, 2024) will include qualitative discussions with general practice teams that will explore invitation letter and poster usage. It is anticipated that the IMP^2^ART-developed patient resources will encourage patients to attend their annual asthma reviews (invitation letters); encourage patients to enquire about asthma plan ownership at their general practice (posters); and equip patients with the knowledge to successfully manage their asthma (information website).

### Conclusions

We conclude that a multi-stage development process, following guidance on how to develop complex interventions to improve health and healthcare (O’Cathain *et al.*, 2019), contributed to the design and development of the patient resources. Theoretical considerations, multidisciplinary team discussions, and the advice of PPI and professional colleagues, informed the initial development; and mixed-method feedback enabled refinement.
